# BAG3 Alleviates Atherosclerosis by Inhibiting Endothelial-to-Mesenchymal Transition via Autophagy Activation

**DOI:** 10.3390/genes13081338

**Published:** 2022-07-26

**Authors:** Hongtao Diao, Kaili Wu, Dingming Lan, Dongwei Wang, Jingjing Zhao, Bingying Huang, Xiaoqi Shao, Ruonan Wang, Huiling Tan, Xinyuan Tang, Meiling Yan, Yue Zhang

**Affiliations:** 1 Guangdong Metabolic Diseases Research Center of Integrated Chinese and Western Medicine, Guangdong TCM Key Laboratory for Metabolic Diseases, Guangdong Pharmaceutical University, Guangzhou 510006, China; diaohongtao@gdpu.edu.cn (H.D.); kl.wu@outlook.com (K.W.); landingming@outlook.com (D.L.); wang1179399063@outlook.com (D.W.); jingjingzhao1022@outlook.com (J.Z.); huangbingying2@outlook.com (B.H.); shaoxiaoqi@gdpu.edu.cn (X.S.); wrn17806065027@outlook.com (R.W.); Tfeiling@outlook.com (H.T.); tangxinyuan9917@outlook.com (X.T.); yanmeiling0122@gdpu.edu.cn (M.Y.); 2 Key Laboratory of Glucolipid Metabolic Disorder, Ministry of Education of China, Guangzhou 510006, China

**Keywords:** atherosclerosis, EndMT, autophagy, CASA complex, BAG3

## Abstract

Atherosclerosis is a chronic systemic inflammatory disease that causes severe cardiovascular events. B cell lymphoma 2-associated athanogene (BAG3) was proven to participate in the regulation of tumor angiogenesis, neurodegenerative diseases, and cardiac diseases, but its role in atherosclerosis remains unclear. Here, we aim to investigate the role of BAG3 in atherosclerosis and elucidate the potential molecular mechanism. In this study, ApoE^−/−^ mice were given a tail-vein injection of BAG3-overexpressing lentivirus and fed a 12-week high-fat diet (HFD) to investigate the role of BAG3 in atherosclerosis. The overexpression of BAG3 reduced plaque areas and improved atherosclerosis in ApoE^−/−^ mice. Our research proves that BAG3 promotes autophagy in vitro, contributing to the suppression of EndMT in human umbilical vein endothelial cells (HUVECs). Mechanically, autophagy activation is mediated by BAG3 via the interaction between BAG3 and its chaperones HSP70 and HSPB8. In conclusion, BAG3 facilitates autophagy activation via the formation of the chaperone-assisted selective autophagy (CASA) complex interacting with HSP70 and HSPB8, leading to the inhibition of EndMT during the progression of atherosclerosis and indicating that BAG3 is a potential therapeutic target for atherosclerosis.

## 1. Introduction

Atherosclerosis, a prevalent chronic vascular disease, is the primary inducement of cardiovascular and cerebrovascular diseases such as coronary heart disease and stroke, which have become the leading causes of death worldwide [1]. Endothelial dysfunction is a risk factor for atherosclerosis and an early predictor of atherosclerosis development [2]. Vascular endothelial cell (EC) injury breaks the integrity and the barrier function of the endothelium, which facilitates the deposition of lipids and leads to atherogenesis [3]. Endothelial-to-mesenchymal transition (EndMT), the process by which ECs undergo a series of molecular events that result in a mesenchymal cell phenotype [4], is characterized by the loss of endothelial markers and functions and the acquisition of mesenchymal markers and functions. During EndMT, ECs increase the secretion of extracellular matrix (ECM) proteins and migratory abilities [5,6]. Previous studies have confirmed that EndMT contributes to the fibrosis of plaque formation and the instability of plaque in the pathobiological development of atherosclerosis [7,8]. As EndMT plays a major role in the atherosclerotic process [9], it is of great significance for the prevention and treatment of atherosclerosis to explore novel and effective targets to alleviate vascular endothelial injury.

BAG3 contains a BAG domain located at the C terminus, a tryptophan–tryptophan (WW) domain at the N-terminus, a proline-rich region (PXXP), two conserved Ile-Pro-Val (IPV) domains and two phosphoserine-containing 14-3-3 binding motifs, which promotes BAG3’s interaction with several proteins and the regulation of many biological pathways [10]. Previous studies have indicated that BAG3 plays an essential role in various cardiovascular diseases (CVDs), such as myocardial hypertrophy, dilated cardiomyopathy and chronic heart failure. In the heart, BAG3 facilitates autophagy by acting as the co-chaperone with heat shock proteins, inhibits apoptosis by binding to B-cell lymphoma 2 and links the thea-adrenergic receptor to the L-type Ca^2+^ channel [11]. In addition, a study has demonstrated that BAG3 is expressed in HUVECs and regulates tumor neoangiogenesis [12]. However, the effect of BAG3 on atherosclerosis remains unclear.

Autophagy is a cellular catabolic system by which cells maintain homeostasis. Autophagy is characterized by the autophagic clearance of dysfunctional organelles, abnormally aggregated proteins, lipid accumulation, and infected pathogens [13,14,15]. The dysregulation of autophagy in ECs was reported to be associated with a variety of pathologic conditions because of the biological significance of autophagy [16,17,18]. The relationship between autophagy activity and the atherosclerotic process illustrates that autophagy in early atheroma lesions may be a transient self-defense mechanism that then declines following prolonged lipid oxidation and oxidative stress [19]. Autophagy emerges as a main protective mechanism in the endothelium [20]. Accumulating evidence indicates that ox-LDL results in atherosclerotic EC injury and promotes atherosclerotic progression, whereas autophagy activation reduces EC injury caused by ox-LDL to alleviate atherosclerosis [21,22,23]. However, the association between autophagy and EndMT has not been identified. The underlying mechanisms for autophagy-induced EndMT still need to be investigated.

The current study aimed to investigate the role of BAG3 in the progress of atherosclerosis and the potential mechanisms. To this end, our findings reveal that BAG3 protects against atherosclerosis by suppressing EndMT with the chaperone-assisted selective autophagy complex triggered by autophagy activation.

## 2. Materials and Methods

### 2.1. Animals and Biochemical Assays

Male ApoE^−/−^ mice were obtained at 6–7 weeks of age from Beijing Huafukang Biotechnology (Beijing, China). The mice were kept under standard animal room conditions with 55% ± 5% humidity and a temperature of 25 ± 1 °C. For BAG3 overexpression, the sequence of LV-BAG3 (NM_013863) and CON335 (negative control) were synthesized and cloned into lentivirus expression vector GV492 (GeneChem Co., Ltd., Shanghai, China). The vector construction and virus packaging were carried out by GeneChem Co., Ltd. The mice were randomly divided into an ND (normal diet) group, an HFD (high-fat diet) group, an HFD + LV-BAG3 group, and an HFD + CON335 group, with 8 animals in each group. They were simultaneously injected with stroke-physiological saline solution, CON335 lentivirus, or BAG3-overexpressing lentivirus (6 × 10^7^ TU/mL) via the tail vein. Then, the animals in the ND group were fed a normal chow diet, while the other animals were fed a high-fat diet (HFD: 20% fat and 1.25% cholesterol) by Xietong Pharmaceutical Bioengineering (Nanjing, China) for 12 weeks to induce atherosclerosis. After successful modeling, the hearts, aortas and blood samples of the mice were collected. All surgeries were conducted under bromoethane anesthesia. Detection of the blood lipid levels was performed according to the manufacturer’s instructions with four indexes of blood lipid assay kits, including a total cholesterol assay kit, a triglyceride assay kit, a low-density lipoprotein cholesterol assay kit, and a high-density lipoprotein cholesterol assay kit (Nanjing Jiancheng Biology Engineering, Nanjing, China).

### 2.2. Atherosclerotic Lesion Assay

After 12 weeks, the aortas and hearts were isolated from the mice. The cardiac tissues were fixed with 4% paraformaldehyde overnight before dehydrating with 15%, and 30% sucrose sequentially. Then, tissues were embedded using the OCT (optimal cutting temperature compound). OCT-embedded cardiac tissues were sectioned into 7 µm thick slices. The frozen sections were used for Oil Red O staining and immunofluorescence experiments. Oil Red O staining of the aorta and aorta root was performed with an Oil red O staining kit (Nanjing Jiancheng Biology Engineering, Nanjing, China) to obtain the lipid deposition. As for the hematoxylin–eosin (H&E) staining assay, tissues were fixed in 4% paraformaldehyde overnight, then embedded with paraffin, and cut into 4 μm sections, which were used for H&E staining assay. H&E staining of the aorta root was performed with an H&E staining kit (Nanjing Jiancheng Biology Engineering, Nanjing, China) to show the atherosclerotic lesions.

### 2.3. Immunohistochemistry

The frozen sections of tissues were washed by phosphate-buffered saline (PBS), then penetrated with 4% Triton X-100 containing 1% BSA for 20 min. After blocking with goat serum for 1 h at room temperature, tissues were incubated in the primary antibodies at 4 °C overnight followed by incubation with Alexa Fluor 488-conjugated secondary antibodies (Invitrogen, Carlsbad, CA, USA) or Alexa Fluor 594-conjugated secondary antibodies (Invitrogen, Carlsbad, CA, USA) for 1 h. The sections were observed under a confocal laser scanning microscope (LSM800, Olympus, Tokyo, Japan).

### 2.4. Cell Culture and Transfection

Human umbilical vein endothelial cells (HUVECs) were purchased from ScienCell Research Laboratories (8000, Sciencell, San Diego, CA, USA) and cultured in the endothelial cell medium (1001, Sciencell, San Diego, CA, USA). For the transfection experiments, BAG3 siRNA, ATG5 siRNA, and HSPB8 siRNA obtained from RIBOBIO Co., Ltd. (Guangzhou, China) were used to knockdown BAG3 and ATG5, and BAG3 pcDNA3.1 obtained from Shanghai Integrated Biotech Solutions Co., Ltd. (Shanghai, China) was used to overexpress BAG3 in the HUVECs. After 7 h of transfection, the medium was replaced by a fresh medium. Then, the HUVECs were stimulated with 100 μg/mL ox-LDL (Dalian Meilun Biotechnology, Dalian, China) for 36 h, 5 μM Apoptozole (ApexBio Technology, Houston, TX, USA) for 18 h or 200 nM rapamycin (Sigma-Aldrich, St Louis, MO, USA) for 24 h, and experiments in vitro were performed post-drug treatment.

### 2.5. Reverse Transcription and Quantitative Real-Time PCR

RNA samples were extracted with TRIzol reagent (Invitrogen, Carlsbad, CA, USA) followed by reverse-transcription with the ReverTra Ace qPCR RT Kit (TOYOBO, Osaka, Japan). Real-time PCR was performed using an SYBR **^®^** Green Realtime PCR Master Mix (TOYOBO, Osaka, Japan) on the PCR System (7500 FAST, ABI, Carlsbad, CA, USA) and the relative levels of mRNAs are calculated according to the values of 2^−^^ΔΔCT^. The sequences of the primers are listed in Table 1.

### 2.6. Western Blot Analysis

The total protein in the HUVECs was extracted with RIPA buffer containing protease inhibitors. After quantification, the protein samples were loaded on 10% or 13% SDS-PAGE gels and transferred to nitrocellulose (NC) membranes followed by blocking with 5% non-fat milk dissolved in PBS for 2 h. The primary antibodies of BAG3 (10599-1-AP, Proteinates’ Group, Inc., Chicago, IL, USA), CD31 (ab24590, Abcam, Cambridge, UK), α-SMA (ab124964, Abcam, Cambridge, UK), LC3 (83506, CST, Danvers, MA, USA), p62 (5114, CST, Danvers, MA, USA), ATG5 (12994, CST, Danvers, MA, USA) and GAPDH (60004-1-Ig, proteinates’ Group, Inc., Chicago, IL, USA) were incubated overnight at 4 °C. After incubation with secondary antibodies, the positive bands were analyzed using Odyssey Infrared Imaging System (LI-COR Biosciences, Lincoln, NE, USA).

### 2.7. Immunocytochemistry

The HUVECs were fixed with 4% paraformaldehyde, and then permeabilized with 0.3% Triton X-100 containing 1% BSA for 20 min, and blocked with goat serum. After blocking, cells were incubated with primary antibodies against CD31 (3528, CST, Danvers, MA, USA), α-SMA (ab124964, Abcam, Cambridge, UK), BAG3 (10599-1-AP, proteinates Group, Inc., Chicago, IL, USA), and HSP70 (66183-1-Ig, ProteinTech Group, Inc., Chicago, IL, USA) overnight at 4 °C. The next day, after incubation with Alexa Fluor 488-conjugated secondary antibodies (Invitrogen, Carlsbad, CA, USA) or Alexa Fluor 594-conjugated secondary antibodies (Invitrogen, Carlsbad, CA, USA) for 50 min, fluorescence was observed using a fluorescence microscope (BX53, Olympus, Tokyo, Japan).

### 2.8. Transwell Assay

Transwell inserts with a 3.0 μm pore size were used for performing cell migration and invasion assays. The cells were resuspended using serum-free medium and the concentration was adjusted to 1 × 10^5^ cells. Medium containing 5% serum was added to each well in 24-well plates and incubated at 37 °C for 24 h in a humidified incubator. The cells remaining on the upper surface of the membrane were removed, and the cells that migrated to the lower surface were fixed in 4% paraformaldehyde for 40 min and stained with 0.1% crystal violet. The migrated cells were counted under an inverted microscope.

### 2.9. GFP-mRFP-LC3II Punctation

The HUVECs were transfected with GFP-mRFP-LC3II lentivirus (Hanbio Biotechnology, Shanghai, China) for 1 h and then the medium was replaced with normal medium. After 36 h, the cells were washed with PBS and viewed under a confocal laser scanning microscope (LSM800, Olympus, Tokyo, Japan). GFP and RFP dots were counted by the manual counting of fluorescent puncta in five high-power fields.

### 2.10. Transmission Electron Microscopy

The cells were obtained and fixed with 2.5% glutaraldehyde at 4 °C overnight. Then, the prepared samples were dehydrated and stained with uranyl acetate and lead citrate, dehydrated, embedded in epoxide resin, and cut into sections. The changes in HUVECs’ microstructure were observed using transmission electron microscopy.

### 2.11. Co-Immunoprecipitation (Co-IP) Analysis

The interactions between endogenous HSP70 or HSPB8 and BAG3 proteins were detected using a Co-IP kit (Thermo Fisher, Waltham, MA, USA). According to the manufacturer’s instructions, 10 μg of antibody against BAG3 (10599-1-AP, ProteinTech Group, Inc., Chicago, IL, USA) was immobilized. For immunoblot analysis, equal amounts of lysed proteins were separated by electrophoresis on a 12% SDS-PAGE gel and transferred to NC membranes. To detect the interactions between HSP70 and HSPB8 and BAG3 protein, the membranes were incubated with antibodies against HSP70 (66183-1-Ig, ProteinTech Group, Inc., Chicago, IL, USA) and HSPB8 (15287-1-AP, ProteinTech Group, Inc., Chicago, IL, USA). The immunoreactive bands were visualized using an Odyssey imaging system (LI-COR Biosciences, Lincoln, NE, USA).

### 2.12. Statistical Analysis

All values are presented as the mean ± S.E.M. Statistical comparisons were performed by Student’s *t*-test between two groups or one-way ANOVA for multiple comparisons. A *p* < 0.05 was considered to indicate a significant difference. The data were analyzed using the GraphPad Prism 7.0 software (GraphPad Software, San Diego, CA, USA).

## 3. Results

### 3.1. Overexpression of BAG3 Reduces Atherosclerotic Lesions

**Figure 1:**
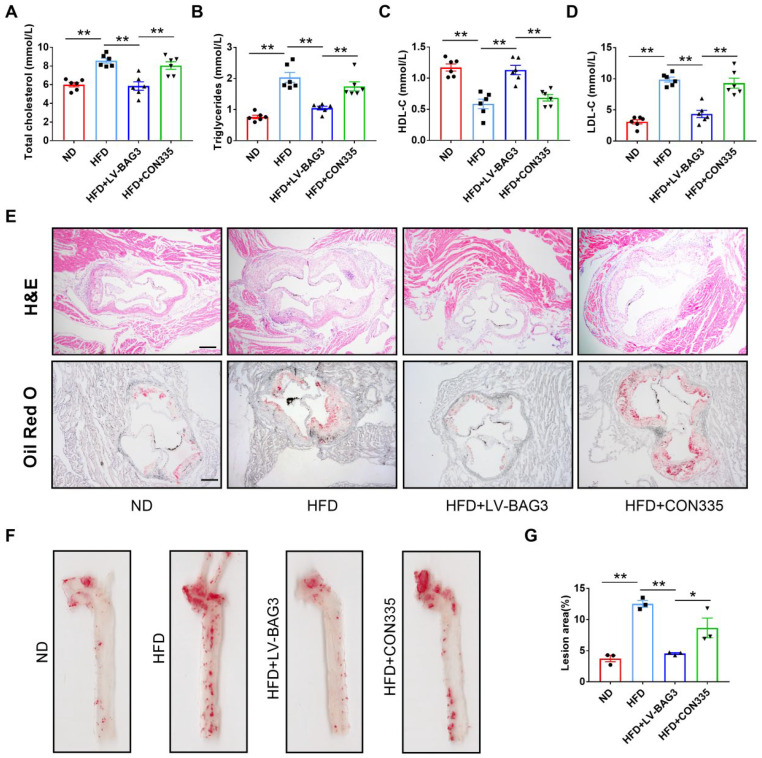
Overexpression of BAG3 reduces atherosclerotic lesions in ApoE^−/−^ mice. (**A**–**D**) The levels of serum TG, TC, HDL-C and LDL-C in mice (*n* = 6). (**E**) Hematoxylin–eosin (H&E) and Oil Red O staining of aortic root sections showing the atherosclerotic lesions and lipid deposition (scale bar indicates 200 μm) (*n* = 3). (**F**,**G**) Representative en face images of Oil Red O staining of aortas; the lesion area of the whole aorta was quantified (*n* = 3). The data are presented as the mean ± S.E.M. * *p* < 0.05, ** *p* < 0.01.

To investigate the role of BAG3 in atherosclerosis, ApoE^−/−^ mice were given a tail-vein injection of LV-BAG3 or CON335 and fed with an HFD for 12 weeks. The overexpression of BAG3 reduced the serum levels of TC, TG, and LCL-C and elevated the level of HDL-C in ApoE^−/−^ mice (Figure 1A–D). In addition, the results of H&E staining and Oil Red O staining revealed that atherosclerosis was successfully induced in the model. We observed a significant increase in lesion size and lipid content in ApoE^−/−^ mice fed with HFD compared to the ND group. Strikingly, the BAG3 overexpression mice showed significantly decreased lesion areas and lipid contents (Figure 1E–G). Taken together, BAG3 overexpression relieved the development of atherosclerosis.

### 3.2. BAG3 Prevents EndMT in the Condition of Atherosclerosis

**Figure 2:**
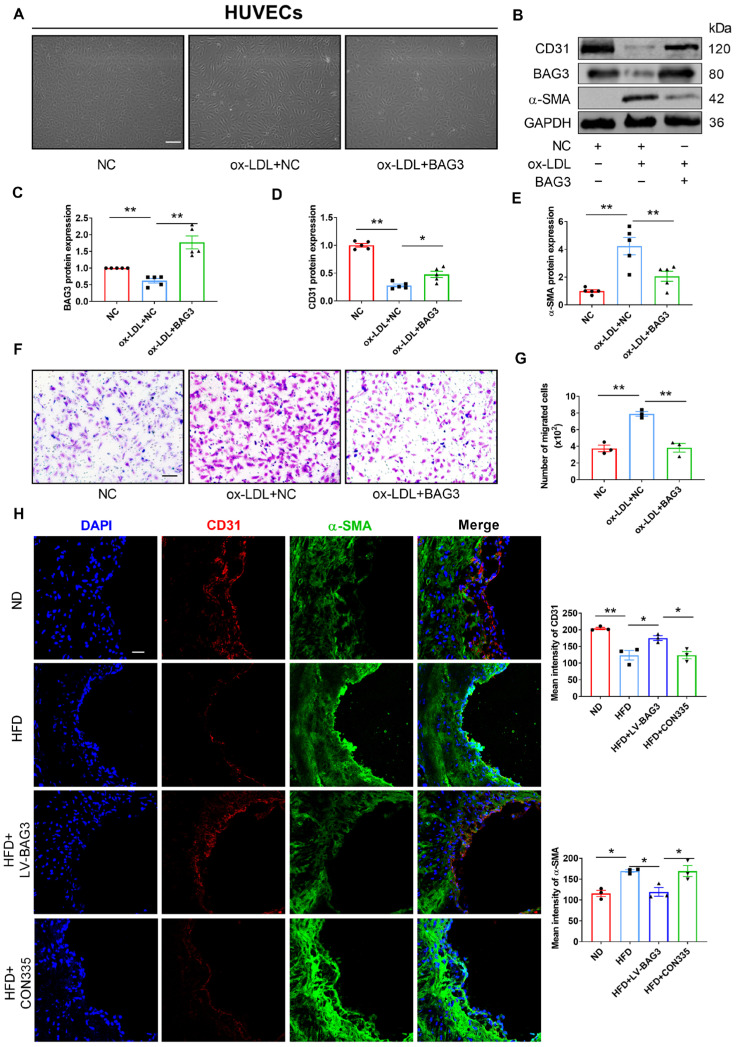
BAG3 prevents EndMT in vivo and vitro. (**A**) Morphological changes post the indicated treatments were observed under the microscope (scale bar indicates 100 µm) (*n* = 3). (**B**–**E**) CD31, BAG3 and α-SMA protein expressions were detected using Western blotting. Fold changes are shown (*n* = 5). (**F**,**G**) Transwell cell invasion assay was performed to analyze the migration ability of cells (scale bar indicates 100 µm) (*n* = 3). (**H**) Representative images of double-fluorescent staining with α-SMA (green) and CD31 (red) in the endothelium. The nuclei were stained blue with DAPI. (scale bar indicates 25 μm) (*n* = 3). The data are presented as the mean ± S.E.M. * *p* < 0.05, ** *p* < 0.01.

To further investigate the effect of BAG3 in the atherosclerotic process, HUVECs were transfected with a negative control (NC) or BAG3-pcDNA3.1 and then treated with oxidized low-density lipoprotein (ox-LDL). The cell morphology of ox-LDL-treated HUVECs was drastically altered and exhibited a spindle-like morphology, whereas HUVECs treated with BAG3-pcDNA3.1 recovered the characteristic cobblestone-like structure, which was nearly identical to normal CD31+ cells (Figure 2A). Meanwhile, Western blot and PCR analyses were performed to detect the protein and mRNA expression of the major genes involved in EndMT. PCR and Western blot analysis revealed a downregulation of ECs markers and upregulation of mesenchymal cell markers in the ox-LDL group, whereas the overexpression of BAG3 inhibited the ox-LDL-induced EndMT (Supplementary Figure S1A–E and Figure 2B–E). Moreover, the Transwell permeability assay demonstrated that the permeability coefficient of BAG3-overexpressed cells was lower than that in the control ones (Figure 2F,G). Furthermore, the results of immunofluorescence staining showed that the expression of endothelial marker CD31 was markedly increased and α-SMA was significantly decreased in ApoE^−/−^ mice with BAG3 lentivirus injection (Figure 2H). The immunocytofluorescence staining of HUEVCs was consistent with the above results (Supplementary Figure S1F). To sum up, the overexpression of BAG3 alleviated EndMT.

### 3.3. BAG3 Prevents EndMT in the Condition of Atherosclerosis

**Figure 3:**
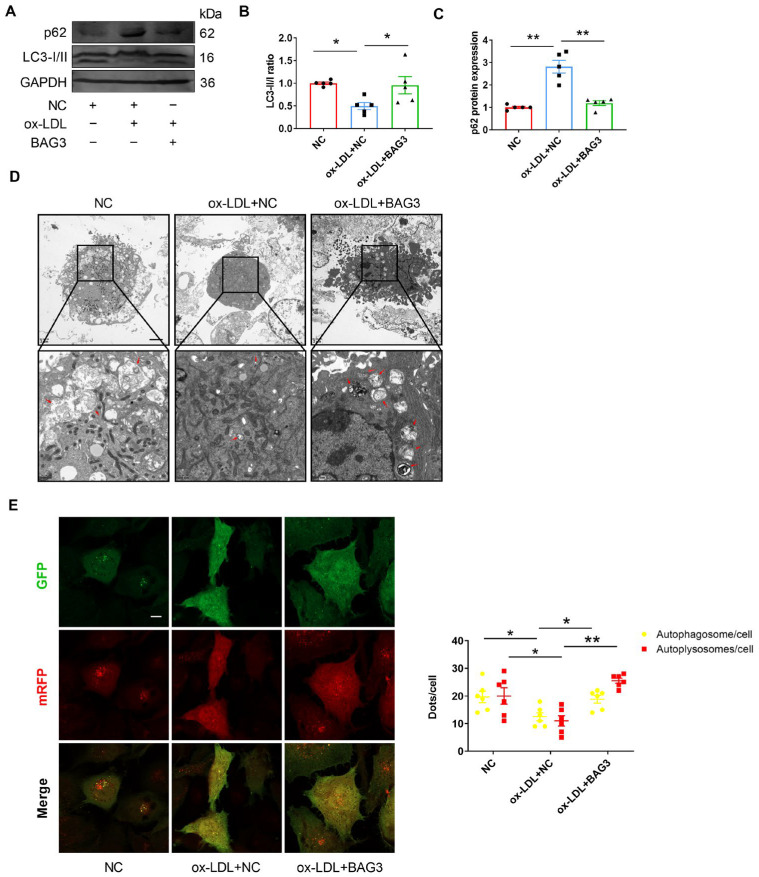
BAG3 positively regulates autophagy in HUVECs. (**A**–**C**) The LC3-II/LC3-I ratio and p62 protein expressions were detected using Western blotting. Fold changes are shown (*n* = 5). (**D**) Electron micrographs of HUVECs submitted to the indicated treatments, including autophagosomes and autolysosomes (red arrow) (scale bar indicates 2 µm and 500 nm) (*n* = 3). (**E**) Representative images and quantification of GFP-mRFP-LC3II puncta in HUVECs. (scale bar indicates 25 µm) (*n* = 5). The data are presented as the mean ± S.E.M. * *p* < 0.05, ** *p* < 0.01.

BAG3 is known to facilitate autophagy in the heart [10,12]. To verify whether BAG3 can regulate autophagy in the vascular endothelium, we treated HUVECs with ox-LDL and BAG3-pcDNA3.1. Regarding autophagy-related proteins, the LC3-II/LC3-I ratio was significantly downregulated while p62 protein expression was increased in ox-LDL-treated HUVECs, and the effect was suppressed upon BAG3 overexpression (Figure 3A–C). In addition, the silencing of BAG3 reduced the number of autophagosomes, while rapamycin rescued the reduction of autophagosomes (Figure 3D). Importantly, autophagic flux in HUVECs was observed by transfection of ad-GFP-mRFP-LC3II. We found that HUVECs with the silencing of BAG3 showed a decreased number of RFP^+^GFP^+^ (autophagosomes) and RFP^+^GFP^−^ (autolysosomes) LC3II puncta compared to the NC group (Figure 3E). These data suggested the importance of BAG3 in autophagy in HUVECs.

### 3.4. Inhibition of Autophagy Induces EndMT in HUVECs

**Figure 4:**
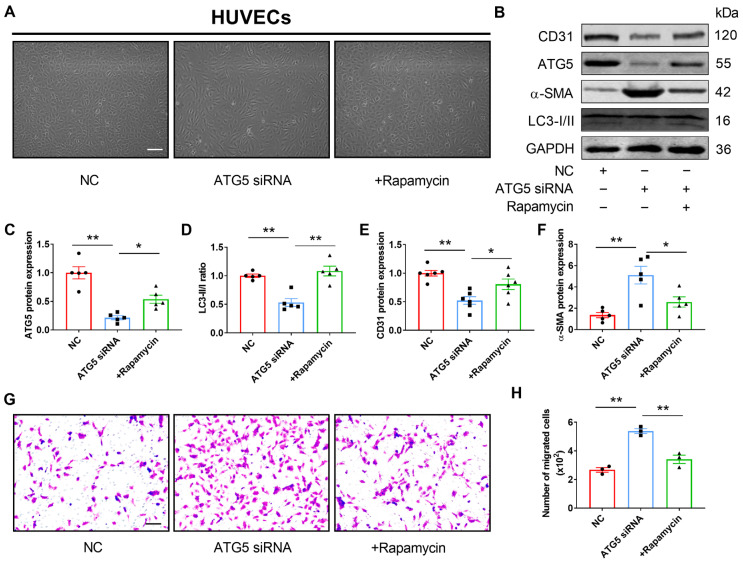
Inhibition of autophagy induces EndMT in HUVECs. (**A**) Morphological changes post the indicated treatments were observed under the microscope (scale bar indicates 100 µm) (*n* = 3). (**B**–**F**) ATG5, CD31, and α-SMA protein expressions and the LC3-II/LC3-I ratio were detected using Western blotting. Fold changes are shown (*n* = 5). (**G**,**H**) Transwell cell invasion assay was performed to analyze the migration ability of cells (scale bar indicates 100 µm) (*n* = 3). The data are presented as the mean ± S.E.M. * *p* < 0.05, ** *p* < 0.01.

To confirm the regulatory effect of autophagy on EndMT in HUVECs, we transfected ATG5 siRNA into HUVECs to block the activation of autophagy. Compared with the NC group, HUVECs with the silence of ATG5 displayed an endothelial cobblestone appearance, which exhibited an elongated mesenchymal morphology (Figure 4A). Meanwhile, we found that the mRNA and protein expressions of endothelial markers were downregulated while mesenchymal markers were upregulated in the ATG5 knockdown HUVECs, and the effect was reversed by rapamycin treatment (Supplementary Figure S2 and Figure 4B–F). The results of the Transwell assay suggested that knockdown of ATG5 increased the permeability of HUVECs, and this variation could be eliminated by the co-application of rapamycin (Figure 4G,H). Collectively, these findings indicated that autophagy inhibits EndMT in HUVECs.

### 3.5. BAG3 Suppresses EndMT by Autophagy

**Figure 5:**
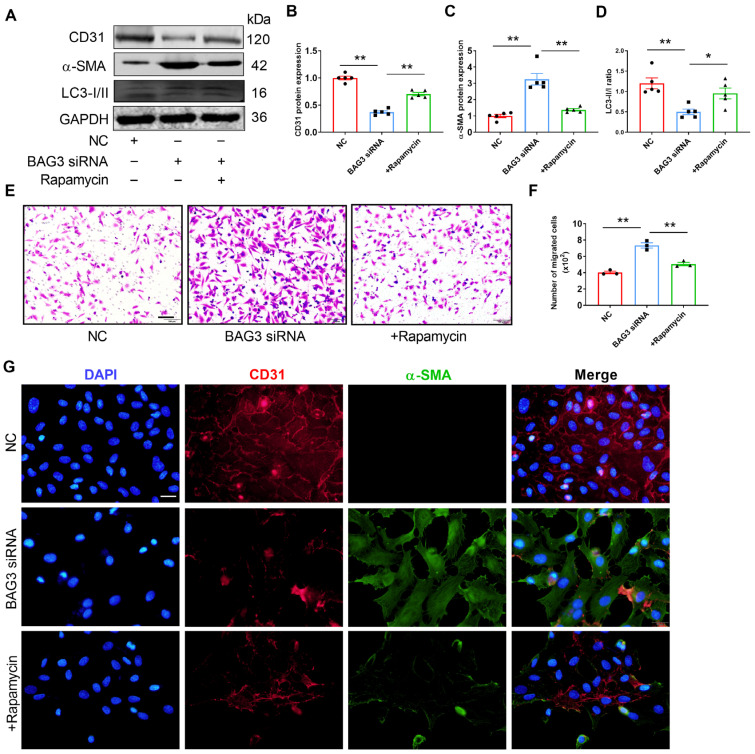
BAG3 suppresses EndMT by inducing autophagy in HUVECs. (**A**–**D**) CD31 and α-SMA protein expressions and the LC3-II/LC3-I ratio were detected using Western blotting. Fold changes are shown (*n* = 5). (**E**,**F**) Transwell cell invasion assay was performed to analyze the migration ability of cells (scale bar indicates 100 µm) (*n* = 3). (**G**) Representative images of double-fluorescent staining with α-SMA (green) and CD31 (red). The nuclei were stained blue with DAPI. (scale bar indicates 50 µm) (*n* = 3). The data are presented as the mean ± S.E.M. * *p* < 0.05, ** *p* < 0.01.

To demonstrate whether BAG3 affects EndMT through autophagy activation, we performed a rescue experiment. The knockdown of BAG3 presented lower levels of EC markers and higher levels of mesenchymal cell markers as shown by PCR, Western blot analysis and immunofluorescent staining. However, the results were reversed in HUVECs after rapamycin treatment. In addition, BAG3 knockdown decreased the ratio of LC3-II to LC3-I, and this effect was suppressed by rapamycin treatment (Supplementary Figure S3 and Figure 5A–D). Meanwhile, the Transwell assay results revealed that BAG3 deficiency increased the migration ability of HUVECs, while rapamycin eliminated this effect (Figure 5E,F). Consistently, immunohistochemical staining revealed an increased EndMT gene signature after the loss of BAG3, which was reversed by rapamycin treatment (Figure 5G). Thus, BAG3 deletion promoted EndMT by autophagy inhibition.

### 3.6. BAG3 Blocks Ox-LDL-Induced EndMT through Formation of CASA Complex with HSP70 and HSPB8

**Figure 6:**
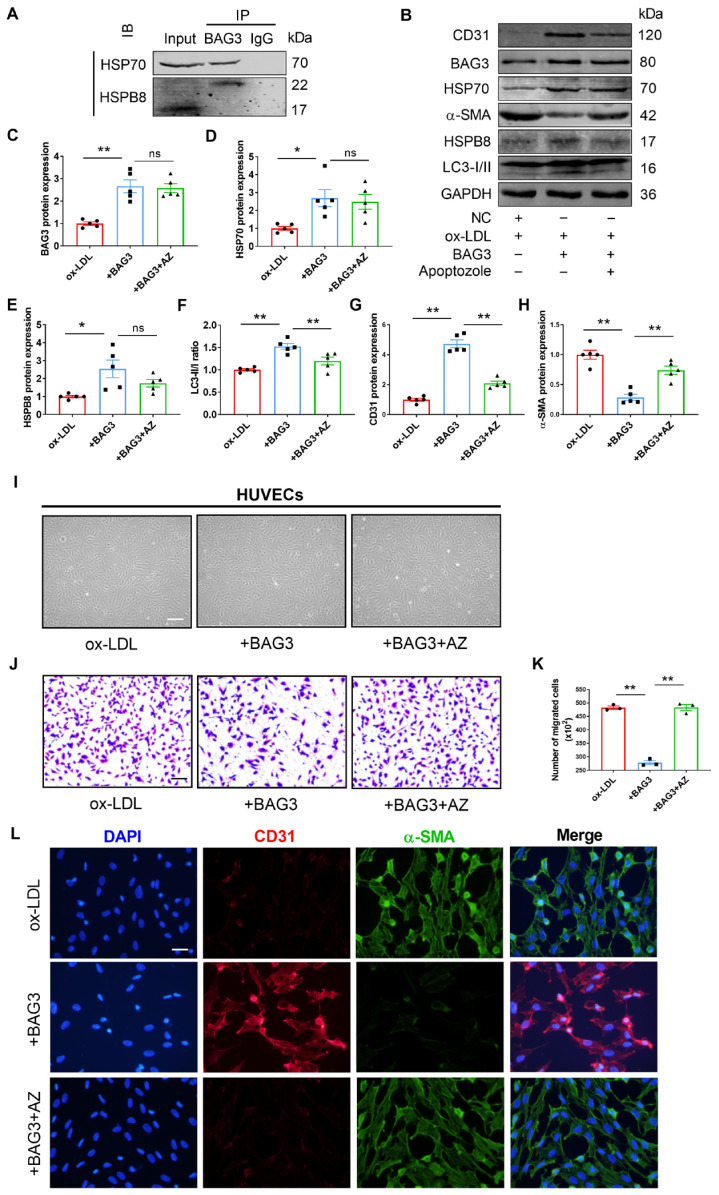
BAG3 blocks ox-LDL-induced EndMT through formation of CASA complex with HSP70 and HSPB8 in HUVECs. (**A**) Co-IP analysis for the interaction of BAG3 with HSP70 or HSPB8. HUVEC extracts were immunoprecipitated with anti-BAG3 antibody and probed with anti-HSP70 or anti-HSPB8 antibody. (*n* = 3). (**B**–**H**) BAG3, HSP70, HSPB8, CD31, and α-SMA protein expressions and the LC3-II/LC3-I ratio were analyzed using Western blotting. Fold changes are shown (*n* = 5). (**I**) Morphological changes post the indicated treatments were observed under a microscope (scale bar indicates 100 µm). (**J**,**K**) Transwell cell invasion assay was performed to analyze the migration ability of cells (scale bar indicates 100 µm). (*n* = 3). (**L**) Representative images of double-fluorescent staining with α-SMA (green) and CD31 (red). The nuclei were stained blue with DAPI (scale bar indicates 50 µm). The data are presented as the mean ± S.E.M. ^ns^ *p* > 0.05, * *p* < 0.05, ** *p* < 0.01.

BAG3 is a member of the co-chaperones with a BAG domain that binds to the N-terminal ATPase domain of heat shock protein 70 (HSP70) [24,25,26]. BAG3 binds to small heat shock proteins (sHSPs), such as HSPB8, via two IPV motifs [27,28,29]. The CASA complex, which is made up of BAG3 and its molecular chaperones HSP70 and HSPB8, has a protective effect on the heart [30]. As mentioned above, BAG3 inhibited EndMT by promoting autophagy. We further investigated whether BAG3 regulates EndMT by activating autophagy through the presence of the CASA complex. Co-IP analysis revealed the interactions between BAG3 and HSP70 or HSPB8 in HUVECs (Figure 6A), indicating the existence of the CASA complex in ECs. BAG3 overexpression-mediated suppression of α-SMA expression, and promotion of the LC3-II/LC3-I ratio, and CD31 expression in HUVECs, which was reversed by treatment with Apoptozole, an inhibitor of the ATPase activity of HSP70. Although the overexpression of BAG3 elevated the expressions of HSPB8, BAG3, and HSP70, the addition of Apoptozole had no influence on their levels (Figure 6B–H). To further understand the role of BAG3 on EndMT in the atherosclerosis model, we used BAG3 pcDNA with and without Apoptozole in ox-LDL treated HUVECs. Observation of the cell morphology observation showed that the BAG3 overexpressed group exhibited the characteristic cobblestone-like morphology as normal HUVECs, while transfected HUVECs became elongated with ox-LDL and additional Apoptozole treatment (Figure 6I). Meanwhile, the Transwell assay results showed that BAG3 overexpression decreased the migration ability of ox-LDL treated HUVECs, while Apoptozole abolished this effect (Figure 6J,K). Consistently, immunohistochemical staining showed an increased EndMT gene signature in ox-LDL treated HUVECs after the overexpression of BAG3, which was reversed by Apoptozole treatment (Figure 6L). Taken together, our results confirmed the interaction between BAG3 and HSP70, and HSPB8 in HUVECs, suggesting that BAG3 rescues EndMT by activating autophagy through the development of the CASA complex.

## 4. Discussion

In the present study, overexpression of BAG3 reduced atherosclerotic lesions in ApoE^−/−^ mice. Thus, we hypothesized that BAG3 plays an important role in the atherosclerotic process. To explore the potential mechanism of BAG3 in atherosclerosis, HUVECs were treated with ox-LDL to induce endothelial injury. The expression of BAG3 decreased in the injured HUVECs induced by ox-LDL stimulation. The knockdown of BAG3 inducing EndMT was attributed to defective autophagy. Mechanically, BAG3 could bind its molecular chaperone HSP70 and HSPB8 to form the CASA complex, which mediated the cargo-selective form of autophagy and allowed the ubiquitination of selected proteins recognized by HSP70 via the CHIP ubiquitin ligase and sequestration by autophagosomes through the LC3 adapter p62/SQSTM in the presence of synaptopodin2 [31], thereby inhibiting EndMT by enhancing autophagy.

Human BAG3 is 575 amino acids in length and has a molecular mass of 74 kDa [32], mainly seen in the cytoplasm [33]. Previous studies have indicated that BAG3 is involved in various cardiovascular diseases, such as myocardial hypertrophy, dilated cardiomyopathy, and chronic heart failure. However, BAG3 has rarely been mentioned in vascular diseases. Falco et al. identified that BAG3 expresses in HUVECs and regulated tumor neoangiogenesis, and therefore, it is a novel target for anti-angiogenic therapies [12]. In recent years, Shasha Yu and Yao Fu have reported that angiotensin II promoted the phenotypic transformation of primary rat VSMCs through the regulation of BAG3 expression [34,35]. Thus, we hypothesized that BAG3 might be a novel therapeutic target in the setting of atherosclerosis. In our study, we found that the expression of BAG3 in HUVECs was downregulated with ox-LDL treatment. In addition, BAG3 overexpression reduced atherosclerotic lesions in ApoE^−/−^ mice, indicating that BAG3 plays an important protective role in atherosclerosis.

Although the development of atherosclerosis involves a variety of cells [36], it is mainly initiated by endothelial cell activation through modified lipids such as oxidized low-density lipoprotein [37]. As a recently recognized type of cellular transdifferentiation, EndMT was first described by Leonard M. Eisenberg in heart development [38], the concept of which was also put forward to explain the pathophysiological process of atherosclerosis from the perspective of cell transdifferentiation [39]. Su et al. reported that ox-LDL could promote Snail, an EndMT transcriptional factor in human aortic endothelial cells (HAECs), in an ox-LDL receptor-dependent manner [40]. In the present study, HUVECs were treated with ox-LDL to determine the atherosclerotic models of endothelial injury. The morphological observation showed that ox-LDL-treated HUVECs were altered and exhibited spindle-like from cobble-like morphology, accompanied by the loss of endothelial cell markers and a gain of mesenchymal cell markers. In addition, their cell migration ability was significantly increased. However, the effect was abolished by the overexpression of BAG3. Thus, the overexpression of BAG3 effectively alleviated ox-LDL-induced injury of HUVECs. The results of the animal experiments were consistent with the results of the cell experiments. Our data suggested that overexpression of BAG3 could inhibit endothelial injury and rescue EndMT in vivo and in vitro, illustrating that BAG3 might be targeted therapeutically to restore atherosclerosis. Several experimental studies have shown that autophagy dysregulation causes a significant increase in cardiovascular diseases, meanwhile, autophagy activation is always associated with the improvement of cardiac and vascular functions [41,42]. Hsieh Paishiun et al. described that knockout of autophagy transcriptional regulator Kruppel-like transcription factor-4 (KLF4) in the endothelium results in a stunted in vivo response to the vasodilator acetylcholine, indicative of endothelial dysfunction through suppressing autophagy [43]. However, autophagy reactivation by rapamycin improves this functional deficit, which denotes the relationship between autophagy and endothelial function [44]. Taken together, these findings strongly suggest that autophagy plays an essential role in maintaining the homeostasis of the cardiovascular system.

Emerging evidence has demonstrated that ox-LDL may induce autophagy dysfunction in ECs [45], while impaired autophagy flux contributes to vascular endothelial dysfunction and atherosclerotic plaque development by regulating lipid metabolism dysfunction [19,46]. Our study confirms autophagic flux impairment and EndMT induced by ox-LDL in HUVECs. Additionally, ox-LDL treatment repressed the expression of endothelium marker CD31 and the ratio of LC3-II/LC3-I and increased the level of mesenchymal marker α-SMA and EC migration. Moreover, we found that the knockdown of ATG5 aggravated EndMT through autophagy repression, which was eliminated by rapamycin-induced autophagy activation. These results indicate that the suppression of EndMT in endothelium was caused by autophagy activation.

As a multifunctional HSP70 co-chaperone and anti-apoptotic protein, BAG3 interacts with the ATPase domain of heat shock protein 70 (HSP70) through the C-terminal BAG domain [47]. Important motifs in the M-domain of BAG3 are two IPV motifs that bind to a series of small HSPs, including HSPB8 [27,48]. HSPB8 acts as a dimer bound to the HSP70 co-chaperone BAG3, a scaffold protein that is also capable of binding to HSP70 (associated with the E3-ligase CHIP) and dynein [49]. Misfolded proteins are engulfed into nascent autophagosomes to be degraded via the chaperone-assisted selective autophagy CASA formed by the two molecules of HSPB8, the HSP70 cochaperone BAG3 and the HSP70 itself [50]. A previous study revealed that cancer therapeutic JG-98, which is toxic for cardiomyocytes, inhibits the BAG3–HSP70 interaction and mitigates tumor growth, also reduced autophagy flux and altered the expression of BAG3 and several binding partners involved in BAG3-dependent autophagy, including HSPB8 [51]. Moreover, BAG3 is critical for the protein turnover of sHSPs via the activation of autophagy in the heart [52]. Our data indicated that BAG3 could form the chaperone-assisted selective autophagy CASA complex with HSP70 and HSPB8 in HUVECs. Moreover, overexpression of BAG3 upregulated the levels of its co-chaperones HSP70 and HSPB8. Formed by these proteins, the CASA complex induced vascular endothelium autophagy activation through the elevated ratio of LC3-II/LC3-I, and the effect was reversed by the ATPase domain inhibitor of HSP70.

In addition, autophagy activation reduced the mesenchymal markers in atherosclerosis models and rescued endothelium dysfunction. Furthermore, the study revealed that BAG3 inhibited endothelial cell injury caused by defective autophagy and EndMT, while EndMT was induced by defective autophagy. These data illustrated that BAG3 prevented endothelial injury by activating autophagy via chaperone-assisted selective autophagy complex formation, thereby helping to improve atherosclerosis. The present study identified the correlation between EndMT and autophagy and clarified that BAG3 could regulate autophagy-induced EndMT by constituting the CASA complex. In the context of atherosclerosis, further work is needed to explore the role of the CASA complex in different types of cells in atherosclerotic lesions, including vascular smooth muscle cells and macrophages. Future studies will need to focus on further unraveling the specific mechanism of the CASA complex in atherosclerotic progression, identifying whether the CASA complex or its component could be a biomarker for the diagnosis of atherosclerosis. Much work remains to be carried out this way.

## 5. Conclusions

In summary, BAG3 mitigates atherosclerosis by suppressing EndMT via synthesizing a CASA complex with HSP70 and HSPB8 to activate autophagy. Our research illustrates that BAG3 has a protective effect against atherosclerosis and provides a new target for treating atherosclerosis.

## Figures and Tables

**Table 1:** Primer sequences used in the present study.

Primers		Sequence
BAG3	Forward	5′-CTCCATTCCGGTGATACACGA-3′
	Reverse	5′-TGGTGGGTCTGGTACTCCC-3′
CD31	Forward	5′-GCAACACAGTCCAGATAGTCGT-3′
	Reverse	5′-GACCTCAAACTGGGCATCAT-3′
VE-cadherin	Forward	5′-GAGCCGCCGCCGCAGGAAG-3′
	Reverse	5′-CGTGAGCATCCAGCAGTGGTAGC-3′
α-SMA	Forward	5′-AGGTAACGAGTCAGAGCTTTGGC-3′
	Reverse	5′-CTCTCTGTCCACCTTCCAGCAG-3′
FSP1	Forward	5′-GTCCACCTTCCACAAGTAC-3′
	Reverse	5′-TGTCCAAGTTGCTCATCAG-3′
ATG5	Forward	5′-TGGACAGTTGCACACACTAGGA-3′
	Reverse	5′-TCAGATGTTCACTCAGCCACTG-3′

## Data Availability

Data are available through the corresponding author.

## Supplementary Materials

The following supporting information can be downloaded at: https://www.mdpi.com/article/10.3390/genes13081338/s1, Figure S1: BAG3 prevents EndMT in vitro; Figure S2: Inhibition of autophagy induces EndMT in vitro; Figure S3: BAG3 suppresses EndMT by inducing autophagy in vitro.

## References

[1] Libby P., Ridker P.M., Hansson G.K. (2011). Progress and challenges in translating the biology of atherosclerosis. Nature.

[2] Gimbrone M.A., Garcia-Cardena G. (2016). Endothelial Cell Dysfunction and the Pathobiology of Atherosclerosis. Circ. Res..

[3] Zhang Y., Qin W., Zhang L., Wu X., Du N., Hu Y., Li X., Shen N., Xiao D., Zhang H. (2015). MicroRNA-26a prevents endothelial cell apoptosis by directly targeting TRPC6 in the setting of atherosclerosis. Sci. Rep..

[4] Li Y., Zhang Y.X., Ning D.S., Chen J., Li S.X., Mo Z.W., Peng Y.M., He S.H., Chen Y.T., Zheng C.J. (2021). Simvastatin inhibits POVPC-mediated induction of endothelial-to-mesenchymal cell transition. J. Lipid Res..

[5] Giordo R., Ahmed Y.M.A., Allam H., Abusnana S., Pappalardo L., Nasrallah G.K., Mangoni A.A., Pintus G. (2021). EndMT Regulation by Small RNAs in Diabetes-Associated Fibrotic Conditions: Potential Link with Oxidative Stress. Front. Cell Dev. Biol..

[6] Hong L., Du X., Li W., Mao Y., Sun L., Li X. (2018). EndMT: A promising and controversial field. Eur. J. Cell Biol..

[7] Chen P.Y., Qin L., Baeyens N., Li G., Afolabi T., Budatha M., Tellides G., Schwartz M.A., Simons M. (2015). Endothelial-to-mesenchymal transition drives atherosclerosis progression. J. Clin. Investig..

[8] Evrard S.M., Lecce L., Michelis K.C., Nomura-Kitabayashi A., Pandey G., Purushothaman K.R., d’Escamard V., Li J.R., Hadri L., Fujitani K. (2016). Endothelial to mesenchymal transition is common in atherosclerotic lesions and is associated with plaque instability. Nat. Commun..

[9] Souilhol C., Harmsen M.C., Evans P.C., Krenning G. (2018). Endothelial-mesenchymal transition in atherosclerosis. Cardiovasc. Res..

[10] Sturner E., Behl C. (2017). The Role of the Multifunctional BAG3 Protein in Cellular Protein Quality Control and in Disease. Front. Mol. Neurosci..

[11] Myers V.D., McClung J.M., Wang J., Tahrir F.G., Gupta M.K., Gordon J., Kontos C.H., Khalili K., Cheung J.Y., Feldman A.M. (2018). The Multifunctional Protein BAG3: A Novel Therapeutic Target in Cardiovascular Disease. JACC Basic Transl. Sci..

[12] Falco A., Festa M., Basile A., Rosati A., Pascale M., Florenzano F., Nori S.L., Nicolin V., Di Benedetto M., Vecchione M.L. (2012). BAG3 controls angiogenesis through regulation of ERK phosphorylation. Oncogene.

[13] Ballabio A., Gieselmann V. (2009). Lysosomal disorders: From storage to cellular damage. Biochim. Biophys. Acta.

[14] Glick D., Barth S., Macleod K.F. (2010). Autophagy: Cellular and molecular mechanisms. J. Pathol..

[15] Lapaquette P., Guzzo J., Bretillon L., Bringer M.A. (2015). Cellular and Molecular Connections between Autophagy and Inflammation. Mediators Inflamm..

[16] Gonzalez C.D., Lee M.S., Marchetti P., Pietropaolo M., Towns R., Vaccaro M.I., Watada H., Wiley J.W. (2011). The emerging role of autophagy in the pathophysiology of diabetes mellitus. Autophagy.

[17] Singh R., Kaushik S., Wang Y., Xiang Y., Novak I., Komatsu M., Tanaka K., Cuervo A.M., Czaja M.J. (2009). Autophagy regulates lipid metabolism. Nature.

[18] Yang L., Li P., Fu S., Calay E.S., Hotamisligil G.S. (2010). Defective hepatic autophagy in obesity promotes ER stress and causes insulin resistance. Cell Metab..

[19] Li W., Sultana N., Siraj N., Ward L.J., Pawlik M., Levy E., Jovinge S., Bengtsson E., Yuan X.M. (2016). Autophagy dysfunction and regulatory cystatin C in macrophage death of atherosclerosis. J. Cell. Mol. Med..

[20] Kluge M.A., Fetterman J.L., Vita J.A. (2013). Mitochondria and endothelial function. Circ. Res..

[21] Wang Y., Che J., Zhao H., Tang J., Shi G. (2019). Paeoniflorin attenuates oxidized low-density lipoprotein-induced apoptosis and adhesion molecule expression by autophagy enhancement in human umbilical vein endothelial cells. J. Cell. Biochem..

[22] Yang Q., Wang C., Jin Y., Ma X., Xie T., Wang J., Liu K., Sun H. (2019). Disocin prevents postmenopausal atherosclerosis in ovariectomized LDLR−/− mice through a PGC-1alpha/ERalpha pathway leading to promotion of autophagy and inhibition of oxidative stress, inflammation and apoptosis. Pharmacol. Res..

[23] Zhou X., Yang J., Zhou M., Zhang Y., Liu Y., Hou P., Zeng X., Yi L., Mi M. (2019). Resveratrol attenuates endothelial oxidative injury by inducing autophagy via the activation of transcription factor EB. Nutr. Metab..

[24] Bracher A., Verghese J. (2015). The nucleotide exchange factors of Hsp70 molecular chaperones. Front. Mol. Biosci..

[25] Briknarova K., Takayama S., Homma S., Baker K., Cabezas E., Hoyt D.W., Li Z., Satterthwait A.C., Ely K.R. (2002). BAG4/SODD protein contains a short BAG domain. J. Biol. Chem..

[26] Takayama S., Xie Z., Reed J.C. (1999). An evolutionarily conserved family of Hsp70/Hsc70 molecular chaperone regulators. J. Biol. Chem..

[27] Fuchs M., Poirier D.J., Seguin S.J., Lambert H., Carra S., Charette S.J., Landry J. (2009). Identification of the key structural motifs involved in HspB8/HspB6-Bag3 interaction. Biochem. J..

[28] Morelli F.F., Mediani L., Heldens L., Bertacchini J., Bigi I., Carra A.D., Vinet J., Carra S. (2017). An interaction study in mammalian cells demonstrates weak binding of HSPB2 to BAG3, which is regulated by HSPB3 and abrogated by HSPB8. Cell Stress Chaperones.

[29] Carra S., Seguin S.J., Lambert H., Landry J. (2008). HspB8 chaperone activity toward poly(Q)-containing proteins depends on its association with Bag3, a stimulator of macroautophagy. J. Biol. Chem..

[30] Mizushima W., Sadoshima J. (2017). BAG3 plays a central role in proteostasis in the heart. J. Clin. Investig..

[31] Ulbricht A., Eppler F.J., Tapia V.E., van der Ven P.F., Hampe N., Hersch N., Vakeel P., Stadel D., Haas A., Saftig P. (2013). Cellular mechanotransduction relies on tension-induced and chaperone-assisted autophagy. Curr. Biol..

[32] Lin H., Koren S.A., Cvetojevic G., Girardi P., Johnson G.V.W. (2022). The role of BAG3 in health and disease: A “Magic BAG of Tricks”. J. Cell Biochem..

[33] Behl C. (2016). Breaking BAG: The Co-Chaperone BAG3 in Health and Disease. Trends Pharmacol. Sci..

[34] Fu Y., Chang Y., Chen S., Li Y., Chen Y., Sun G., Yu S., Ye N., Li C., Sun Y. (2018). BAG3 promotes the phenotypic transformation of primary rat vascular smooth muscle cells via TRAIL. Int. J. Mol. Med..

[35] Yu S., Chen Y., Chen S., Ye N., Li Y., Sun Y. (2018). Regulation of angiotensin II-induced B-cell lymphoma-2-associated athanogene 3 expression in vascular smooth muscle cells. Mol. Med. Rep..

[36] Yuan Y., Xu L., Geng Z., Liu J., Zhang L., Wu Y., He D., Qu P. (2021). The role of non-coding RNA network in atherosclerosis. Life Sci..

[37] Dahlof B. (2010). Cardiovascular disease risk factors: Epidemiology and risk assessment. Am. J. Cardiol..

[38] Eisenberg L.M., Markwald R.R. (1995). Molecular regulation of atrioventricular valvuloseptal morphogenesis. Circ. Res..

[39] Wesseling M., Sakkers T.R., de Jager S.C.A., Pasterkamp G., Goumans M.J. (2018). The morphological and molecular mechanisms of epithelial/endothelial-to-mesenchymal transition and its involvement in atherosclerosis. Vascul. Pharmacol..

[40] Su Q., Sun Y., Ye Z., Yang H., Li L. (2018). Oxidized low density lipoprotein induces endothelial-to-mesenchymal transition by stabilizing Snail in human aortic endothelial cells. Biomed. Pharmacother..

[41] Ren J., Yang L., Zhu L., Xu X., Ceylan A.F., Guo W., Yang J., Zhang Y. (2017). Akt2 ablation prolongs life span and improves myocardial contractile function with adaptive cardiac remodeling: Role of Sirt1-mediated autophagy regulation. Aging Cell.

[42] Mattison J.A., Wang M., Bernier M., Zhang J., Park S.S., Maudsley S., An S.S., Santhanam L., Martin B., Faulkner S. (2014). Resveratrol prevents high fat/sucrose diet-induced central arterial wall inflammation and stiffening in nonhuman primates. Cell Metab..

[43] Hsieh P.N., Zhou G., Yuan Y., Zhang R., Prosdocimo D.A., Sangwung P., Borton A.H., Boriushkin E., Hamik A., Fujioka H. (2017). A conserved KLF-autophagy pathway modulates nematode lifespan and mammalian age-associated vascular dysfunction. Nat. Commun..

[44] Wang Q., Wu S., Zhu H., Ding Y., Dai X., Ouyang C., Han Y.M., Xie Z., Zou M.H. (2017). Deletion of PRKAA triggers mitochondrial fission by inhibiting the autophagy-dependent degradation of DNM1L. Autophagy.

[45] Bravo-San Pedro J.M., Kroemer G., Galluzzi L. (2017). Autophagy and Mitophagy in Cardiovascular Disease. Circ. Res..

[46] Zhou Y.H., Tang Y.Z., Guo L.Y., Zheng L.L., Zhang D., Yang C.Y., Wang W. (2022). Overexpression of sFlt-1 represses ox-LDL-induced injury of HUVECs by activating autophagy via PI3K/AKT/mTOR pathway. Microvasc. Res..

[47] Sondermann H., Scheufler C., Schneider C., Hohfeld J., Hartl F.U., Moarefi I. (2001). Structure of a Bag/Hsc70 complex: Convergent functional evolution of Hsp70 nucleotide exchange factors. Science.

[48] Rauch J.N., Tse E., Freilich R., Mok S.A., Makley L.N., Southworth D.R., Gestwicki J.E. (2017). BAG3 Is a Modular, Scaffolding Protein that physically Links Heat Shock Protein 70 (Hsp70) to the Small Heat Shock Proteins. J. Mol. Biol..

[49] Arndt V., Dick N., Tawo R., Dreiseidler M., Wenzel D., Hesse M., Furst D.O., Saftig P., Saint R., Fleischmann B.K. (2010). Chaperone-assisted selective autophagy is essential for muscle maintenance. Curr. Biol..

[50] Carra S., Seguin S.J., Landry J. (2008). HspB8 and Bag3: A new chaperone complex targeting misfolded proteins to macroautophagy. Autophagy.

[51] Martin T.G., Delligatti C.E., Muntu N.A., Stachowski-Doll M.J., Kirk J.A. (2022). Pharmacological inhibition of BAG3-HSP70 with the proposed cancer therapeutic JG-98 is toxic for cardiomyocytes. J. Cell. Biochem..

[52] Inomata Y., Nagasaka S., Miyate K., Goto Y., Hino C., Toukairin C., Higashio R., Ishida K., Saino T., Hirose M. (2018). Bcl-2-associated athanogene 3 (BAG3) is an enhancer of small heat shock protein turnover via activation of autophagy in the heart. Biochem. Biophys. Res. Commun..

