# Supplementary material for: BAG3 Alleviates Atherosclerosis by Inhibiting Endothelial-to-Mesenchymal Transition via Autophagy Activation
Source: Genes (Basel). 2022 Jul 26;13(8):1338. doi: 10.3390/genes13081338 (PMC9332509; doi:10.3390/genes13081338)
Supplement: Supplementary file 1 [file genes-13-01338-s001.zip › genes-1839485-supplementary.pdf]

Supplemental materials

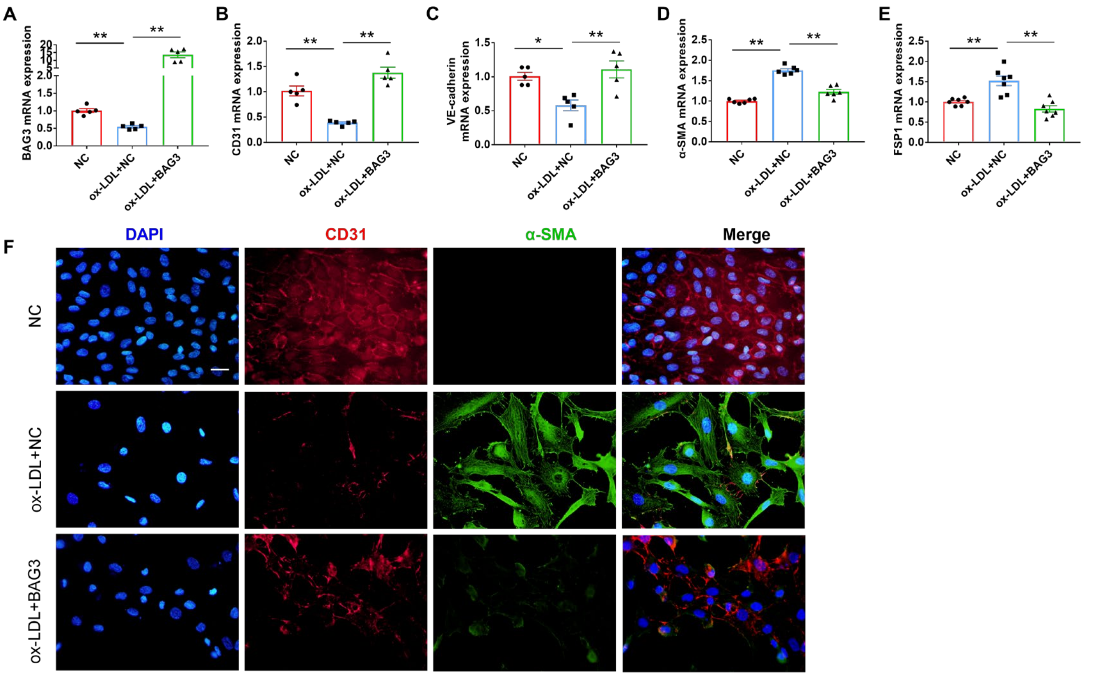

Figure S1. BAG3 prevents EndMT in vitro. (**A-E**) BAG3, CD31, VE-cadherin, α-SMA and FSP1 mRNA expressions were analyzed by RT-PCR, normalized to GAPDH. Fold changes are shown (n=5). (**F**) Representative images of double-fluorescent staining with CD31 (red) and α-SMA (green). The nuclei were stained blue with DAPI. Immunofluorescence signals of CD31 and α-SMA were quantified (Scale bar indicates 50 μm) (n=3). The data are presented as the mean ± S.E.M. $^{*}P < 0.05$, $^{**}P < 0.01$.

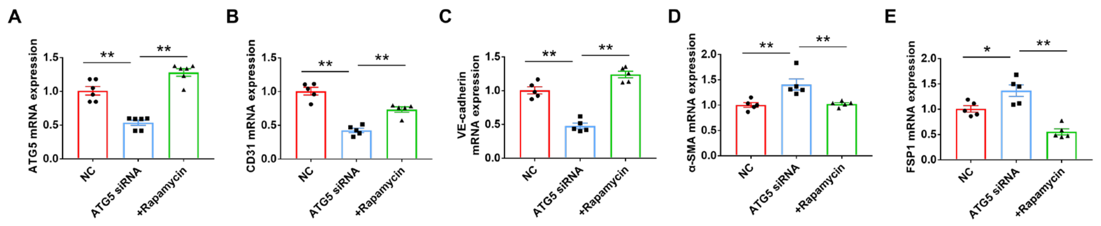

Figure S2. Inhibition of autophagy induces EndMT in vitro. (**A-E**) ATG5, CD31, VE-cadherin, α-SMA and FSP1 mRNA expressions were analyzed by RT-PCR, normalized to GAPDH. Fold changes are shown (n=5) The data are presented as the mean ± S.E.M. $^{*}P < 0.05$, $^{**}P<0.01$.

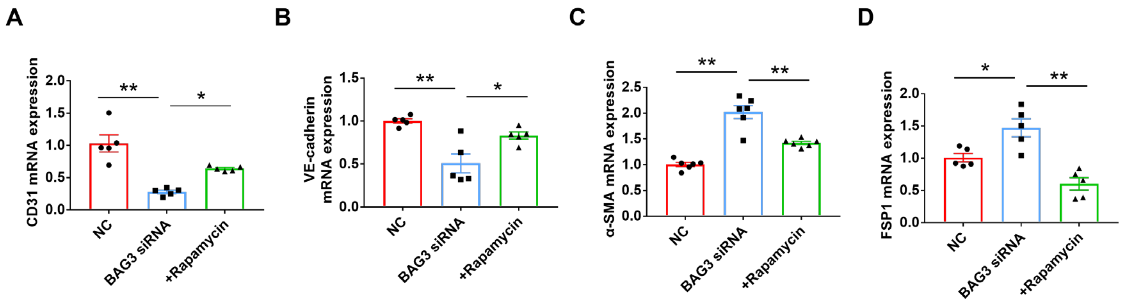

Figure S3. BAG3 suppresses EndMT by inducing autophagy in vitro. (**A-D**) CD31, VE-cadherin, α-SMA and FSP1 mRNA expressions were analyzed by RT-PCR, normalized to GAPDH. Fold changes are shown (n=5). The data are presented as the mean ± S.E.M. $^{*}P < 0.05$, $^{**}P < 0.01$.
